# A Multi-Center Trained Residual Neural Network for Robust Classification of Atrial High-Rate Episodes in Remotely Monitored Pacemakers and Defibrillators

**DOI:** 10.3390/s26072241

**Published:** 2026-04-04

**Authors:** Lars van Krimpen, Arlene John, Anand Thiyagarajah, Tanner Carbonati, Benjamin Sacristan, Karim Benali, Antoine Da Costa, Pierre Mondoly, Rémi Chauvel, Romain Eschalier, Josselin Duchateau, Remi Dubois, Sylvain Ploux, Pierre Bordachar, Marc Strik

**Affiliations:** 1 Cardio-Thoracic Unit, Bordeaux University Hospital (CHU), F-33600 Pessac-Bordeaux, France; 2 Institut Hospitalo-Universitaire L'institut Des Maladies Du Rythme Cardiaque (LIRYC), Electrophysiology and Heart Modeling Institute, Fondation Bordeaux Université, F-33600 Pessac-Bordeaux, France; 3 Biomedical Signals and Systems Group, Twente University, 7522 NB Enschede, The Netherlands; 4 Centre for Heart Rhythm Disorders, Adelaide University, Adelaide, SA 5000, Australia; 5 Saint-Etienne University Hospital Center, Saint-Etienne University, F-42100 Saint-Etienne, France; 6 Department of Cardiology, University Hospital Rangueil, F-31400 Toulouse, France; 7 Cardiology Department, Centre Hospitalier Universitaire Clermont-Ferrand, F-63000 Clermont-Ferrand, France; 8 Institut Pascal, SIGMA Clermont, Centre National de la Recherche Scientifique (CNRS), Université Clermont Auvergne, Centre Hospitalier Universitaire Clermont-Ferrand, F-63000 Clermont-Ferrand, France

**Keywords:** remote monitoring, atrial high-rate episodes, pacemakers, implantable cardiac defibrillators, residual network, deep learning, atrial fibrillation, oversensing

## Abstract

**Highlights:**

**What are the main findings?**

- The presented residual network successfully classifies atrial high-rate episodes of remotely monitored pacemakers and implantable cardiac defibrillators.
- Trusting the model predictions with probabilities higher than 95% would reduce 91.9% of the classification workload while maintaining very high sensitivity for AT/AF.

**What are the implications of the main findings?**

- Remote monitoring of CIEDs can benefit from deep learning models through automated episode review.
- AI can decrease remote monitoring workload by the automatic archiving of known false positive episodes and alerting for noise episodes.

**Abstract:**

Remote monitoring of pacemakers and defibrillators increases patient safety but also increases clinical workload. Review of atrial high-rate episodes is particularly demanding as episodes can contain atrial tachycardia or atrial fibrillation (AT/AF), noise, or far-field oversensing (FFO). Automatic review of atrial high-rate episodes by an Artificial Intelligence (AI) model can decrease the workload of remote monitoring, provided it maintains high sensitivity for true atrial tachycardia. A residual network is trained using a center-level fourfold cross validation. The four resulting models achieved a precision of 97.2–99.4% for AT/AF, 93.1–97.7% for noise, and 75.4–94.4% for FFO, while maintaining high sensitivity 98.9–99.3% for AT/AF. The four models were combined through averaging prediction probabilities to create an ensemble model. Thresholding ensemble predictions with probability > 95% resulted in a robust ensemble model that made only two errors (<0.1%) after reviewing 3925 episodes (91.9%) of the total 4271 episodes. This shows how AI models can reliably assist in remote monitoring. Future research should be aimed at classification models for other episode types and clinical validation of AI models to assist remote monitoring of pacemakers and defibrillators.

## 1. Introduction

Annually, more than 650,000 patients are implanted with a pacemaker or implantable cardiac defibrillator (ICD) in Europe [1]. Implanted patients need monitoring to detect device- or cardiac-related problems, which may be performed through in-clinic follow-up or via remote monitoring. Since its introduction, the use of remote monitoring has increased in popularity due to its additional advantages over sporadic in-clinic follow-up. Remote monitoring of pacemakers and ICDs allows faster intervention [2], reduces patient morbidity and mortality, lowers healthcare costs, and increases quality of life [3,4].

Despite these benefits, the surge in remotely monitored patients and associated transmissions exponentially increased workload in healthcare [5]. Reviewing transmissions is time-consuming and the number of episodes requiring intervention is low; 73–90% of the transmissions are non-actionable [6]. In this context, a pivotal role is recognized for Artificial Intelligence (AI) models to reduce workloads by filtering remote monitoring alerts before they reach the healthcare professional [5].

To date, the application of AI in remote monitoring of cardiac implantable electronic devices has been limited [7]. Deep learning classification models in remote monitoring of cardiac devices are mainly used to reduce false positive alerts of implantable loop recorders [7,8]. In pacemakers and ICDs, we have shown that two-dimensional convolutional neural network models accurately discriminate non-sustained ventricular tachycardias (NSVTs) from noise in NSVT episodes [9]. Despite this success the use of AI for atrial high-rate episode (AHRE) classification remains unexplored.

AHREs alert a remote monitoring center when atrial tachycardia or atrial fibrillation (AT/AF) is suspected, allowing rapid initiation of anticoagulation therapy and a reduction in the risk of stroke [10,11]. However, AHREs often contain false positive (FP) alarms caused by noise or far-field oversensing (FFO) on the atrial channel. Automatic, reliable verification of these episodes by cloud-based deep learning models could improve diagnostic accuracy, reduce the need to analyze every episode, leading to lowered work burden of remote monitoring staff, and increase patient safety. Therefore, the aim of this study is to create a robust deep learning model for classification of AHREs of pacemakers and ICDs.

In this work we present a robust residual deep neural network to classify AHREs of Biotronik pacemakers and ICDs based on a center-level cross validation. During the fourfold cross validation the model is trained on different training data due to different configurations of the data of four hospitals. The resulting four models are combined using soft voting to create an ensemble model to reduce model variance due to center-specific bias in training data. Evaluation of the ensemble model on a fifth-center dataset demonstrated robust classification of AHREs.

## 2. Materials and Methods

### 2.1. Data Acquisition

This study used ‘AT’ and ‘Atrial Monitoring’ episodes of remotely monitored patients with a Biotronik (Biotronik SE & Co. KG, Berlin, Germany) pacemaker or ICD from five different hospitals. Episodes between 2013 and 2021 were collected from Bordeaux, Bayonne, Clermont Ferrand, and Toulouse, resulting in 7131 episodes (726 patients), 1022 episodes (118 patients), 1858 episodes (237 patients), and 739 episodes (67 patients), respectively. The Saint Etienne hospital provided 4271 episodes (116 patients) from 2013 to 2025. Lastly, 7380 of Biotronik ‘Periodic EGM’ episodes containing sinus rhythm, used in our earlier work, were also included in this study [12]. The research reported in this paper adhered to the Helsinki Declaration, data was de-identified prior to analysis, and the ethics review board of Bordeaux University approved the study under reference CER-BDX 2023–46.

**Figure 1:**
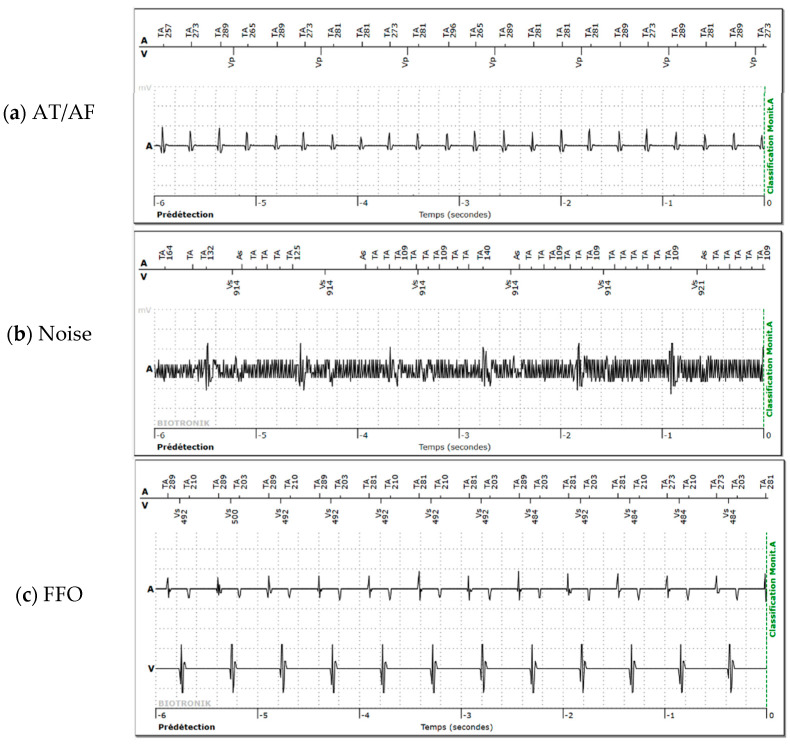
Examples of atrial high-rate episodes found on the Biotronik remote monitoring website containing (**a**) true atrial tachycardia (AT/AF), (**b**) noise, and (**c**) far-field oversensing (FFO). The atrial (A) and ventricular (V) intracardiac electrograms are displayed in seconds ( Temp (secondes) ) before the device detects (Prédétection) an atrial monitoring episode (Classification Monit. A). Depicted atrial and ventricular markers (in French) on the marker channels are ‘TA’ for atrial tachycardia marker, ‘AS’ for atrial sensing marker, ‘Vp’ for ventricular pacing marker, and ‘Vs’ for ventricular sensing marker. Most markers are accompanied by the interval (in milliseconds) to the previous marker.

The AHREs were blindly labeled by two remote monitoring experts as AT/AF, noise, or FFO. Figure 1 shows examples of AHREs containing AT/AF, noise, or FFO. When an episode showed both true AT/AF and noise or oversensing, the true AT/AF class was prioritized. A third remote monitoring expert adjudicated disagreements between the two reviewers.

The atrial intracardiac electrogram (EGM), ventricular EGM, and corresponding markers were extracted from the last ten seconds of each episode using Python 3.12. EGM signals were normalized and marker information was stored in a ten-second binarized signal with a one at the time of a marker and zeros elsewhere. Signals shorter than ten seconds were zero-padded (sample frequency of 128 Hz) at the beginning of the signal to reach ten seconds. Episodes without a ventricular channel were given a ten-second signal of zeros.

### 2.2. Data Augmentation

Training data was enlarged through data augmentation. The augmentation techniques were derived from commonly used ECG augmentation techniques [13]. Augmentation techniques used included: (a) blanking a randomly chosen 2 s window of the episode, (b) randomly scaling the EGM of each channel by a factor of 0.4–1.2, (c) inverting the EGM amplitude of the signals, (d) shifting the EGM signals 0.4–1.5 s, and (e) exchanging the first and second temporal halves of each EGM signal. These augmentation methods were applied to all noise and FFO episodes, and to twenty percent of the AT/AF episodes (4% for each augmentation technique). The augmented data was only used for training the model to distinguish AT/AF, noise, and FFO, and not during pretraining.

### 2.3. Model Design

A custom, one-dimensional residual network (1D-ResNet) model was chosen due to its strong performance in electrocardiogram classification [14] and was created using TensorFlow version 2.20.0. The 1D-ResNet was preferred because its residual connections improve gradient flow during training, mitigating the vanishing gradient problem that is commonly encountered in deep neural networks [15]. Furthermore, the cloud-based implementation intent of the classification model does not require a sequential approach like a long short-term memory model. The 1D-ResNet used atrial and ventricular EGM signals as input. Optimal model structure for classifying AT/AF, noise, and FFO was determined with the data of Bayonne, Bordeaux, Clermont, and Toulouse.

**Figure 2:**
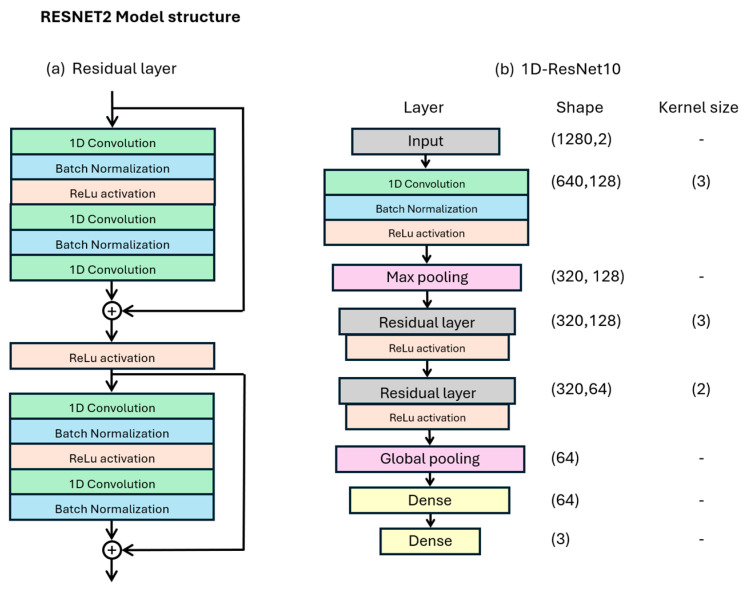
The model structure of the one-dimensional residual neural network (1D-ResNet10) used in this study.

The model structure and hyperparameters were determined prior to the fourfold cross validation using two grid searches. The grid searches aimed to find the optimal model structure (amount of residual blocks, filters, layers per residual block, and kernel sizes) and model regularization-/hyperparameters (batch normalization, dropout layers, batch size, learning rate). Finally, additional manual tuning of hyperparameters resulted in the 1D-ResNet10 model in Figure 2. The first 1D convolution, batch normalization, ReLu activation layer, and max pooling layer extract the important features of the signal into a temporally smaller signal of 320 datapoints. Next, two residual layers consisting of two residual blocks each further extract features from the signals. Lastly, a global pooling layer and two dense layers combine features towards the final predictions for each class.

### 2.4. Model Training and Testing with Multi-Center Fourfold Cross Validation

A hospital-level fourfold cross validation (4FCV) (pre)trained, validated, and tested the predefined 1D-ResNet10 model. The predefined hyperparameters were fixed during the 4FCV. The hospital-level 4FCV resulted in four 1D-ResNet10 models based on different training, validation, and test sets, as displayed in Table 1. In order to have enough patients in the test set of each fold, the data of Bordeaux was split (on the patient-level with class stratification) and the Bayonne data was merged with the data of Toulouse.

During each fold of the 4FCV, the three datasets were split at the patient-level with class rectification to form the training (80%) and validation (20%) set. The 1D-ResNet10 was pretrained to distinguish sinus rhythm (periodic EGM episodes) from AT/AF in the AHREs of the train set. There was no patient overlap between the sinus rhythm episodes and the validation or test set. After pretraining, the weights of all layers except the last dense layer were saved and used to train the 1D-ResNet10 model to classify AT/AF, noise, and FFO. The model was trained with the categorical focal loss function, Adam optimizer with a learning rate of 0.001, and batch size of 64. Training was stopped when model performance did not improve for five epochs or a total of 50 epochs was reached. The model with the highest class-weighted F2-score was saved, which emphasized high sensitivity for the AT/AF class. After the model was trained on the training and validation set, the model was tested on the test set of that fold.

### 2.5. External Testing and Ensemble Model

The four models created during each fold of the 4FCV were tested on the external Saint Etienne test set. Furthermore, the four models were combined through ensemble learning to create a more robust model. Soft voting was used for ensemble learning, meaning that the output probability of each class was averaged across all four models. The final ensemble classification was the class with the highest average probability. Soft voting was chosen to preserve class probabilities and to enable evaluation of ensemble model performance at different probability thresholds.

### 2.6. Outcomes

Model performance was evaluated using the precision, recall, specificity, and F2-score, which can be calculated using Equations (1)–(4) and the true positive (TP), false positive (FP), true negative (TN), and false negative (FN) model predictions. The F2-score gives greater weight to recall than precision, because it is clinically relevant to achieve near-perfect sensitivity for AT/AF, a condition associated with stroke.

(1) $$\mathrm{Precision}\;(\%)=\frac{TP}{TP+FN}\;*\;100$$

(2) $$\mathrm{Recall}\;(\%)=\frac{TP}{TP+FP}\;*\;100$$

(3) $$\mathrm{Specificity}\;(\%)=\frac{TN}{TN+FP}\;*\;100$$

(4) $$F2-\mathrm{score}\;(\%)=\frac{5}{\frac{1}{Precision}+\frac{4}{Recall}}\;*\;100$$

Model performance was also calculated for predictions with probabilities of at least 90% and 95%. These probability thresholds could be relevant for clinical implementation of the model by only trusting model predictions with high probability.

Patient-level performance of the 1D-Resnet10 was calculated by using the predictions of the four models of the 4FCV on their test sets (see Table 1), and the predictions of the ensemble model on the external Saint Etienne test set. The noise and FFO episodes were provided by an insufficient number of patients to calculate patient-level performance from a single test set. Bootstrapping with 1000 iterations was used to estimate performance and 95% confidence intervals. During bootstrapping patients were selected with replacement, and for each patient one episode of each available class was randomly selected. This preserved class balance and provided enough patients for performance estimation on the noise and FFO class.

Lastly, saliency maps using Gradient-weighted Class Activation Mapping (Grad CAM++) were used to obtain insight in important parts of the signal for the (ensemble) model to classify the episode and see if patterns could be translated to clinically relevant patterns [16]. The saliency maps of the four models from 4FCV were normalized and averaged to create the Grad CAM++ maps of the ensemble model.

## 3. Results

### 3.1. Data Overview

**Figure 3:**
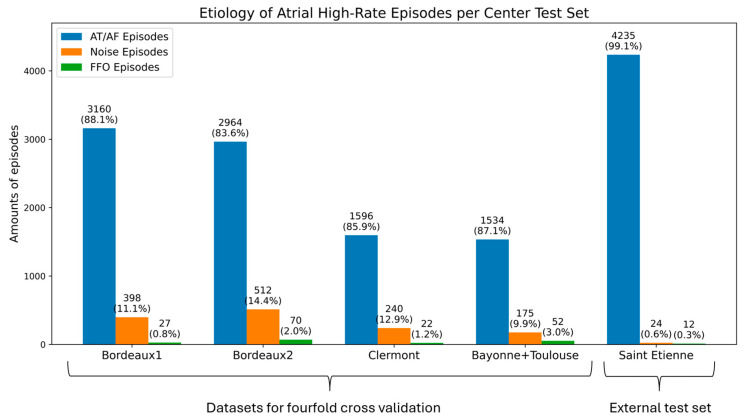
The amount of atrial tachycardia and atrial fibrillation (AT/AF), noise, and far-field oversensing (FFO) episodes across the datasets of different centers.

Figure 3 shows the class distribution among the different datasets used. For all datasets the AT/AF class was the majority class, noise was the second-largest class and FFO episodes form the minority class. The median amount of episodes per patient across all datasets was six (range: 1–1283) for AT/AF, five (range: 1–25) for noise and six (range: 1–25) for FFO. The dataset of Saint Etienne has a low amount of noise and FFO episodes compared to the other datasets due to a high burden of AT/AF episodes (six patients provided 3125 AT/AF episodes).

### 3.2. Model Performance

**Figure A1:**
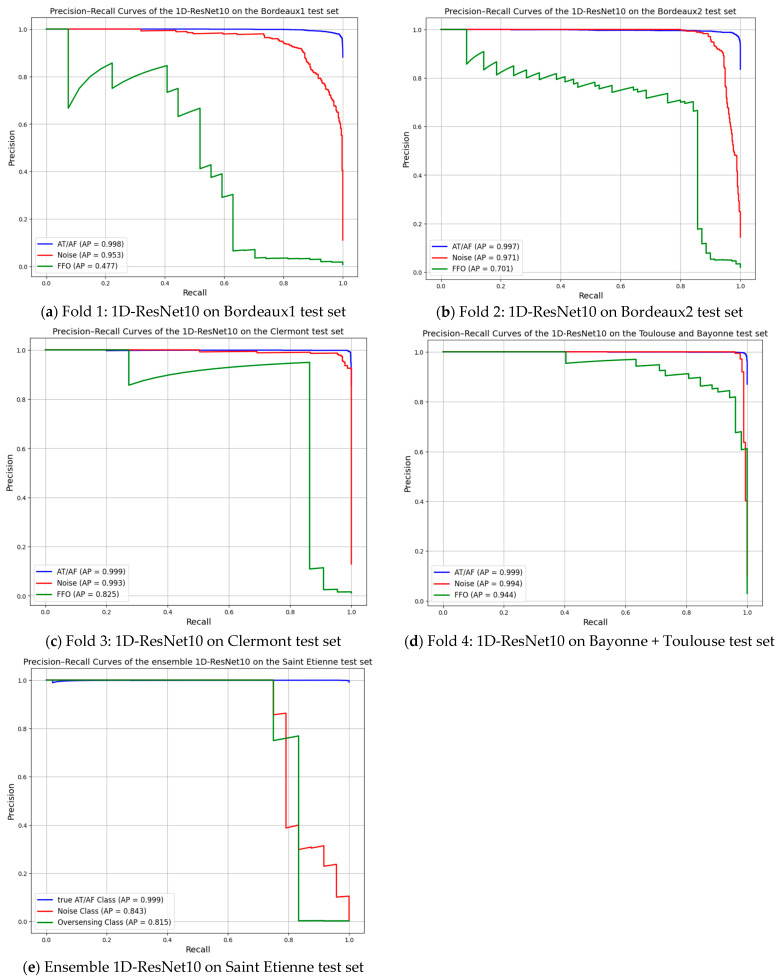
The precision–recall curves (PRC) and average precision (AP) values of the one-dimensional residual network models (1D-ResNet10) for each class. For each fold of the fourfold cross validation, the model performance on the test set is shown in (**a**–**d**). The performance of the ensemble model on the external Saint Etienne test set is shown in (**e**).

Table 2 shows the performance of the model of each fold from the 4FCV. All models had the highest precision and recall for the AT/AF class. The high recall for AT/AF resulted in lower specificity for this class and higher specificity for noise and FFO. All models had the lowest precision and recall for the FFO episodes. The precision–recall curves in Figure A1a–d in Appendix A also reflect superior performance for the AT/AF class, which is followed by the noise and FFO class across all four models. Model performance for all classes increased when predictions with probability of at least 90% and 95% were selected. The number of incorrect predictions of each model decreased to <1% if only predictions with at least 95% probability were selected. At this probability at least 87.7% of all episodes were included and a minimum AT/AF sensitivity of 99.8% was achieved by all models.

Table 3 shows the performance of the four models from the 4FCV and the ensemble model on the dataset of Saint Etienne. Each individual model of the 4FCV achieves a high precision and recall for AT/AF, but classification performance for noise and FFO varies across models of each fold. Performance for the noise and FFO episodes stabilize after thresholding episodes at 95% probability. The ensemble model outperforms each individual model at the prediction probability threshold of 95%, making only two errors by classifying two FFO episodes as AT/AF.

Across all test sets there were 1155 patients with AT/AF episodes, 160 patients with noise episodes and 30 patients with FFO. Patient-level performance of the five models resulted in a recall of 98.3% (95% CI 97.5–99.1) for AT/AF, 91.0% (95% CI 86.9–95.0) for noise, and 57.1% (95% CI 40.0–73.3) for FFO. Patient-level specificity was 86.1% (95% CI 82.1–90.5) for AT/AF, 98.9% (95% CI 98.2–99.4) for noise, and 99.5% (95% CI 99.0–99.8) for FFO.

### 3.3. Gradient-Weighted Class Activation Mapping

**Figure 4:**
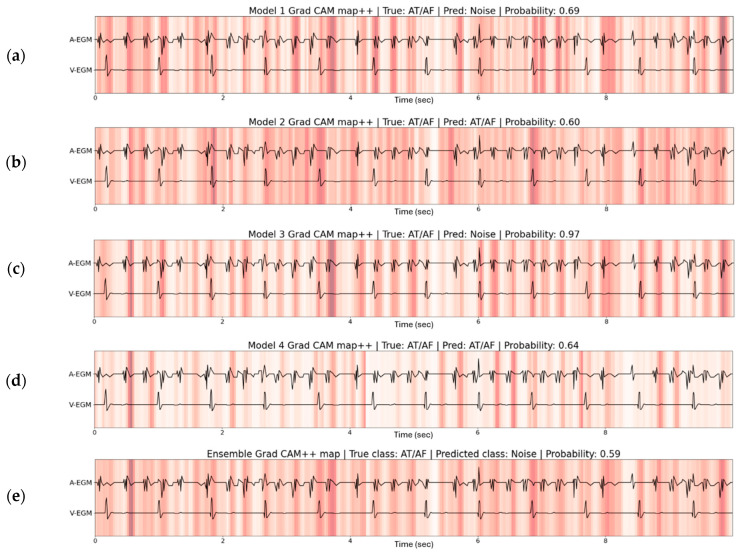
The Grad CAM++ map of the four models from the fourfold cross validation (**a**–**d**) and the ensemble model (**e**) for an AT/AF episode predicted as noise. The Grad CAM++ map is shown for the normalized cardiac electrograms of the atrial (A-EGM) and ventricular (V-EGM) channel. Models 2 and 4 focus on atrial and ventricular activations with similar morphology, possibly to look for non-conducted atrial activations. Models 1 and 3 focus both on high-frequent atrial morphologies and classify the episode as noise. The ensemble model Grad CAM++ map shows the important parts of the model for all four models. Furthermore, the high probability for the noise prediction of model 3 is reduced by the probabilities of the other three models, which led to a probability of 59% for the noise prediction of the ensemble model.

Figure 4 shows the Grad CAM++ plots of the four models of the 4FCV and the ensemble model on the same AHRE containing AT/AF. Two models in Figure 4a,c classify the model as noise and both highlight high frequent signals on the atrial EGM. The models in Figure 4b,d classify the episode as AT/AF and seem to focus on parts of the signals following atrial activations to possibly look for ventricular conduction. The ensemble model Grad CAM++ map is the average map of the four models and shows how the high-frequent signals and post-atrial activation signals are important. The ensemble prediction of the model is noise with a probability of 0.59.

**Figure 5:**
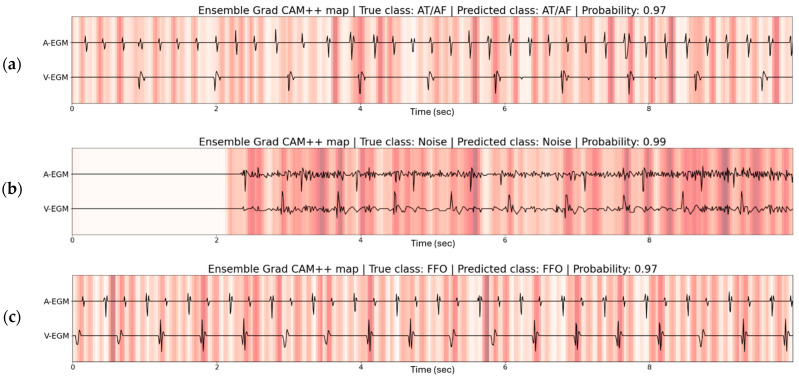
Examples of the Grad CAM++ map of the ensemble model for three correctly classified episodes of (**a**) AT/AF, (**b**) noise, and (**c**) far-field oversensing (FFO). Parts of the normalized atrial and ventricular intracardiac EGMs that were most important to the model for its prediction are given a darker shade on the Grad CAM++ map.

Figure 5 shows the ensemble Grad CAM++ maps for correctly classified AHREs. The correct AT/AF prediction in Figure 5a highlights parts of the signal following atrial activations, possibly to look for atrioventricular conduction and a consequent ventricular activation. Figure 5b highlights high-frequent parts of the atrial and ventricular EGM for classification of a noise episode. Lastly, Figure 5c displays the importance of ventricular activations with similar morphology and the signal following the ventricular activation. The model focusses on the far-field R-wave for this FFO episode.

## 4. Discussion

Our work presents for the first time how AI can automatically label AHREs transmitted by Biotronik pacemakers and ICDs. Through a multi-center cross validation approach, a robust 1D-ResNet10 ensemble model is created with near-perfect sensitivity for AT/AF. Thresholding ensemble model predictions at >95% probability further improves performance and illustrates how the model could be clinically implemented.

### 4.1. Model Performance

The different 1D-ResNet10 models of the folds from 4FCV were all able to differentiate AT/AF, noise, and FFO in the test set of that fold. Furthermore, Table 2 shows that all models were highly sensitive for AT/AF, which is clinically desirable to not miss any cases of AT/AF, as this arrhythmia is associated with stroke [10,11]. Patient-level performance of the models demonstrated similarly high recall for AT/AF.

Performance on the noise and FFO class varies among the different models of each fold, as shown in Table 2. The small number of episodes in the dataset of each center likely underrepresents the diversity of noise and FFO morphologies. Consequently, some models may encounter unseen types of noise or FFO in their test set, leading to decreased performance for these classes due to the center-specific sampling bias. The sampling bias is also seen in Table 3, where models of different folds perform differently on the noise and FFO class of the same external test set. Even though performance on noise and FFO is difficult to assess, due to the low prevalence of these classes in the external test set, lower performance for noise and FFO compared to AT/AF remains evident.

Ensemble learning and thresholding of the prediction probability are used to obtain a robust model for AHRE classification. By taking the average prediction probability of the models and having a probability threshold at >95%, all models need to predict with high probability before an ensemble prediction passes the probability threshold. The effectiveness of this method is shown in Figure 4, where the four individual models had different predictions and the model of fold 3 had an incorrect prediction with high probability. The resulting ensemble prediction was still incorrect, but would not pass the probability threshold and would require review by remote monitoring staff. A prediction probability threshold of 95% is adequate for the ensemble model based on Table 2. At this threshold, the models in Table 2 show a lower error rate and higher sensitivity for AT/AF while at least 87% of the predictions still exceed the threshold. Table 3 displays how the ensemble model outperforms each individual model of the cross validation for predictions with >95% probability. The ensemble ResNet predicts 3935 of 4271 episodes (91.9%) with >95% probability, and only makes two errors (<0.1%) while maintaining 100% sensitivity for AT/AF. The two errors of the ensemble model included FFO episodes that were interpreted as AT/AF. The episodes both showed slightly varying FFO signals which the model interpreted as AT/AF signals. The error rate of the ensemble model shows how the model can be reliable to automatically classify a majority of the AHREs while leaving low-probability predictions for review by a healthcare professional.

The Grad CAM++ maps of the ensemble model in Figure 5 show the highlighted parts of signals for correct predictions of AT/AF, noise, and FFO. The highlighted signals show patterns for each class that are clinically relevant. High-frequent atrial noise is highlighted for noise episodes, morphologically similar ventricular activations and the far-field over sensed R-waves are captured for FFO, and absence of consistent atrioventricular conduction is highlighted for atrial fibrillation. This indicates that the ensemble model focusses on clinically relevant patterns and supports the validity of the model.

### 4.2. Limitations

The class imbalance in the datasets limits the model performance on the minority classes, especially on the FFO class. The performance of all models in Table 2 is lowest for the FFO class, due to the low number of episodes containing FFO in all datasets. Pretraining and the use of data augmentation techniques increased model performance on this class, but more FFO episodes are needed to further improve model performance on FFO. Clinically, FFO episodes are encountered in a small number of patients. Observation of FFO in the clinic will lead to correcting the device programming, for example by extending the far-field protection window, which explains the low incidence of episodes containing FFO. Episodes of FFO can lead to inappropriate mode switches to asynchronous modes. These mode switches are rarely symptomatic and can be considered as a benign pollution of remote monitoring. Therefore, although model performance for FFO needs to improve, clinical consequences are limited when FFO is missed. Lastly, FFO episodes classified as AT/AF could in theory lead to inappropriate initiation of anticoagulation therapy. However, manual verification of the episodes should precede initiation of any medical therapy, including anticoagulation. In the long run, AI adjudication of noise/FFO episodes labeled by the device as AF will decrease the inappropriate initiation of anticoagulation therapy.

### 4.3. Artificial Intelligence Models in Remote Monitoring

Currently, Artificial Intelligence models that assist remote monitoring of cardiac implantable electronic devices (CIEDs) only exist for implantable loop recorders (ILRs). Several vendors have cloud-based models to filter out false positive alerts [17,18,19,20]. These studies report a reduction in false positive alerts of 41.6–72.6% while maintaining a 95.6–99.4% sensitivity for true AT/AF. These studies also showed how reducing the false positive alert burden across all types of ILR alarms resulted in a 42.8–85% reduction in remote monitoring workload. In our external Saint Etienne test set, trusting predictions with >95% probability of the presented ensemble 1D-ResNet10 model would reduce the remote monitoring workload by 92% while maintaining a 100% sensitivity for AT/AF. This seems to be at least as good as the AI models for the ILRs episodes and demonstrates that our ensemble model is ready for real-world evaluation.

### 4.4. Clinical Integration of Artificial Intelligence

Artificial Intelligence assistance for remote monitoring of CIEDs will become necessary to keep the benefits of remote monitoring while managing increasing workload. The latest expert opinion recommended three full-time healthcare professionals per thousand remotely monitored patients [5]. Performing remote monitoring according to this recommendation is rarely seen in practice due to human resource challenges.

We believe AI assistance can and should be available to all without extra cost, focusing on transparency, reliability, and good clinical integration. Direct integration of AI models in CIEDs faces memory and energy constraints due to the low-power hardware of these devices. Therefore, we believe that near-future integration of AI models in remote monitoring will be cloud-based. Cloud-based AI assistance could classify incoming remote monitoring alarms and filter out episodes that are predicted with high-probability. The ability to verify AI filtered episodes should remain, with the healthcare professional always retaining responsibility for clinical decisions. Episodes predicted with lower probability should still be adjudicated by the healthcare professional and could be used to further train the model. Lastly, documentation of model development and clinical testing of the model should be clear in order to trust the model for clinical usage.

## 5. Conclusions

The presented ensemble residual network classifies AHREs in true AT/AF, noise, and FFO while maintaining particular high sensitivity for true AT/AF. Probability thresholding of predictions shows how the ensemble model can reliably assist in remote monitoring by classifying 92% of the episodes with <0.1% error rate and leaving low-probability predictions for review by a healthcare professional. Future work should be aimed at clinical validation of the presented 1D-ResNet10 model and development of other classification models for different remote monitoring alarms.

## Figures and Tables

**Table 1:** The configuration of data from the different hospitals to form the training, validation, and the test set for each fold of the fourfold cross validation.

Fold of Cross Validation	Training and Validation Set	Test Set
1	Bordeaux2, Clermont, Bayonne + Toulouse	Bordeaux1
2	Bordeaux1, Clermont, Bayonne + Toulouse	Bordeaux2
3	Bordeaux1, Bordeaux2, Bayonne + Toulouse	Clermont
4	Bordeaux1, Bordeaux2, Clermont	Bayonne + Toulouse

**Table 2:** Performance of the models from the center-level fourfold cross validation at different prediction probability levels. Model performance is expressed in precision, recall, and specificity for the classes atrial fibrillation/atrial tachycardia (AT/AF), noise (N), and far-field oversensing (FFO).

Test Set	Probability Threshold for Predictions	Incorrect Predictions (*n*)	Precision (%)			Recall (%)			Specificity (%)		
			AT/AF	N	FFO	AT/AF	N	FFO	AT/AF	N	FFO
Bordeaux1	-	116/3585	97.24	93.12	78.57	99.14	81.66	40.74	79.06	99.25	99.92
	90%	46/3318	98.83	98.07	100	99.83	88.81	18.18	86.20	99.84	100
	95%	28/3143	99.17	98.19	-	99.86	93.53	-	90.04	99.86	100
Bordeaux2	-	110/3546	97.70	94.70	75.41	98.68	90.82	65.71	88.14	99.14	99.57
	90%	44/3310	98.80	98.87	80.00	99.68	94.59	66.67	93.09	99.82	99.85
	95%	30/3190	99.06	99.52	85.71	99.89	96.10	54.54	94.32	99.93	99.94
Clermont	-	23/1858	99.37	95.12	94.44	99.25	97.5	77.27	96.18	99.26	99.95
	90%	7/1792	99.81	98.64	92.31	99.74	100	80.00	98.71	99.81	99.94
	95%	6/1747	99.80	99.03	92.31	99.80	100	80.00	98.64	99.87	99.94
Bayonne + Toulouse	-	22/1761	99.22	97.71	88.00	99.35	97.71	84.62	94.71	99.75	99.65
	90%	3/1654	99.80	100	100	100	98.72	94.74	98.29	100	100
	95%	2/1560	99.86	100	100	100	98.59	100	98.65	100	100

**Table 3:** Performance of the 1D-ResNet10 models from the fourfold cross validation and the ensemble model on the external test set Saint Etienne at different prediction probability levels. Model performance is expressed in precision, recall, and specificity for the classes atrial fibrillation/atrial tachycardia (AT/AF), noise (N), and far-field oversensing (FFO).

Model	Probability Threshold for Predictions	Incorrect Episodes (*n*)	Precision (%)			Recall (%)			Specificity (%)		
			AT/AF	N	FFO	AT/AF	N	FFO	AT/AF	N	FFO
Fold 1	-	25/4271	99.81	51.43	100	99.60	75.00	83.33	77.78	99.60	100
	90%	5/4096	99.90	94.12	100	99.98	88.89	80.00	85.71	99.98	100
	95%	3/3904	99.95	93.33	100	99.97	100	71.43	90.48	99.97	100
Fold 2	-	31/4271	99.81	47.73	100	99.46	87.50	58.33	77.78	99.46	100
	90%	7/4141	99.90	85.71	100	99.93	94.74	66.67	85.71	99.93	100
	95%	4/4038	99.93	94.74	100	99.98	100	66.67	88.89	99.98	100
Fold 3	-	58/4271	99.86	28.77	100	98.77	87.50	75.00	83.33	99.78	100
	90%	30/4119	99.93	41.30	100	99.34	100	70.00	89.66	99.34	100
	95%	22/4022	99.92	48.65	100	99.52	100	66.67	88.89	99.53	100
Fold 4	-	24/4271	99.79	59.38	80.00	99.65	79.17	66.67	75.00	99.69	99.95
	90%	4/3980	99.92	92.31	100	99.997	100	66.7	85.71	99.97	100
	95%	4/3625	99.92	88.89	100	99.97	100	62.5	81.25	99.97	100
Ensemble model	-	36/4271	99.79	41.30	100	99.36	79.17	66.67	75.00	99.36	100
	90%	2/4107	99.95	100	100	100	100	75.00	92.31	100	100
	95%	2/3925	99.95	100	100	100	100	75.00	90.00	100	100

## Data Availability

The datasets presented in this article are not readily available because the datasets are part of an ongoing study.

## Abbreviations

The following abbreviations are used in this manuscript:

AHRE	Atrial High-Rate Episode
AI	Artificial Intelligence
AT/AF	Atrial Tachycardia/Atrial Fibrillation
AUROC	Area Under the Receiver Operating Characteristic Curve
CIED	Cardiac Implantable Electronic Device
EGM	Intracardiac Electrogram
FFO	Far-Field Oversensing
FP	False Positive
FN	False Negative
4FCV	Fourfold Cross Validation
Grad CAM	Gradient-weighted Class Activation Mapping
ICD	Implantable Cardiac Defibrillator
ILR	Implantable Loop Recorder
N	Noise
NSVT	Non-Sustained Ventricular Tachycardia
ResNet	Residual Network
TP	True Positive

## Appendix A

**Table A1:** Performance of the models from the center-level fourfold cross validation at different prediction confidence levels. Model performance is expressed in negative predictive value and area under the receiver operating characteristic (AUROC) curve for the classes atrial fibrillation/atrial tachycardia (AT/AF), noise (N), and far-field oversensing (FFO).

Test Set	Probability Threshold for Predictions	Incorrect Predictions (*n*)	Negative Predictive Value (%)			AUROC (%)		
			AT/AF	N	FFO	AT/AF	N	FFO
Bordeaux1	-	116/3585	92.56	97.74	99.55	98.5	99.3	92.6
	90%	46/3318	98.08	98.95	99.73			
	95%	28/3143	98.19	99.49	99.71			
Bordeaux2	-	110/3546	92.93	98.46	99.31	98.6	99.0	95.2
	90%	44/3310	98.07	99.13	99.70			
	95%	30/3190	99.31	99.39	99.69			
Clermont	-	23/1858	95.45	99.63	99.73	99.4	99.9	94.3
	90%	7/1792	98.29	100	99.83			
	95%	6/1747	98.64	100	99.83			
Bayonne + Toulouse	-	22/1761	95.56	99.75	99.53	99.6	99.9	99.8
	90%	3/1654	100	99.87	99.94			
	95%	2/1560	100	99.86	100			

**Table A2:** Performance of the 1D-ResNet10 models from the fourfold cross validation and the ensemble model on the external test set Saint Etienne at different prediction confidence levels. Model performance is expressed in negative predictive value and area under the receiver operating characteristic (AUROC) curve for the classes atrial fibrillation/atrial tachycardia (AT/AF), noise (N), and far-field oversensing (FFO).

Model	Probability Threshold for Predictions	Incorrect Episodes (*n*)	Negative Predictive Value (%)			AUROC (%)		
			AT/AF	N	FFO	AT/AF	N	FFO
Fold 1	-	25/4271	99.81	51.43	100	99.9	77.9	83.4
	90%	5/4096	99.90	94.12	100			
	95%	3/3904	99.95	93.33	100			
Fold 2	-	31/4271	99.81	47.73	100	99.9	85.0	75.2
	90%	7/4141	99.90	85.71	100			
	95%	4/4038	99.93	94.74	100			
Fold 3	-	58/4271	99.86	28.77	100	99.9	51.4	75.3
	90%	30/4119	99.93	41.30	100			
	95%	22/4022	99.92	48.65	100			
Fold 4	-	24/4271	99.79	59.38	80.00	99.9	79.5	68.6
	90%	4/3980	99.92	92.31	100			
	95%	4/3625	99.92	88.89	100			
Ensemble model	-	36/4271	99.79	41.30	100	99.9	84.3	81.5
	90%	2/4107	99.95	100	100			
	95%	2/3925	99.95	100	100			
